# Identification of Potential Clusters of Signs and Symptoms to Prioritize Patients' Eligibility for AADCd Screening by 3-OMD Testing: An Italian Delphi Consensus

**DOI:** 10.1155/2024/1023861

**Published:** 2024-04-09

**Authors:** Carlotta Spagnoli, Roberta Battini, Filippo Manti, Duccio Maria Cordelli, Andrea Pession, Melissa Bellini, Andrea Bordugo, Gaetano Cantalupo, Antonella Riva, Pasquale Striano, Marco Spada, Francesco Porta, Carlo Fusco

**Affiliations:** ^1^Child Neurology and Psychiatry Unit, AUSL-IRCCS di Reggio Emilia, Reggio Emilia, Italy; ^2^Department of Developmental Neuroscience, IRCCS Stella Maris, Pisa, Italy; ^3^Child Neuropsychiatric Unit, Human Neuroscience Department, Sapienza University of Rome, Rome, Italy; ^4^IRCCS Istituto delle Scienze Neurologiche di Bologna, UOC Neuropsichiatria dell'Età Pediatrica, Bologna, Italy; ^5^Department of Medical and Surgical Sciences, Alma Mater Studiorum, University of Bologna, Bologna, Italy; ^6^Pediatric Oncology & Hematology Unit ‘Lalla Seràgnoli', IRCCS Azienda Ospedaliero-Universitaria di Bologna, Bologna, Italy; ^7^Pediatrics and Neonatology Unit, Guglielmo da Saliceto Hospital, AUSL di Piacenza, Piacenza, Italy; ^8^Inherited Metabolic Diseases Unit and Regional Centre for Newborn Screening, Diagnosis and Treatment of Inherited Metabolic Diseases and Congenital Endocrine Diseases, Azienda Ospedaliera Universitaria Integrata, Verona, Italy; ^9^Innovation Biomedicine Section, Department of Engineering for Innovation Medicine, University of Verona, Verona, Italy; ^10^Child Neuropsychiatry Unit and Center for Research on Epilepsy in Pediatric Age (CREP), University Hospital of Verona (full member of the European Reference Network EpiCARE), Verona, Italy; ^11^Pediatric Neurology and Muscular Diseases Unit, IRCCS Istituto Giannina Gaslini, Genova, Italy; ^12^Department of Neurosciences, Rehabilitation, Ophthalmology, Genetics, Maternal and Child Health, University of Genova, Genova, Italy; ^13^Department of Pediatrics, AOU Città della Salute e della Scienza di Torino, University of Torino, Turin, Italy; ^14^Department of Pediatrics, Metabolic Diseases, AOU Città della Salute e della Scienza, University of Torino, Turin, Italy

## Abstract

**Introduction:**

AADCd is an ultrarare, underdiagnosed neurometabolic disorder for which a screening test (3-OMD dosing on dried blood spot (DBS)) and targeted gene therapy (authorized in the EU and the UK) are available. Therefore, it is mandatory to raise awareness of presenting symptoms and signs among practitioners. Delivering scientifically sound information to promote screening of patients with the correct cluster of symptoms and signs would be critical.

**Materials and Methods:**

In light of the lack of sound evidence on this issue, expert opinion level of evidence was elicited with the Delphi method. Fourteen steering committee members invited a panel of 29 Italian experts to express their opinions on a series of crucial but controversial topics related to using 3-OMD DBS as a screening method in AADCd. Clusters of symptoms and signs were divided into typical or atypical, depending on age groups. Inclusion in newborn screening programs and the usefulness of a clinical score were investigated. A five-point Likert scale was used to rate the level of priority attributed to each statement.

**Results:**

The following statements reached the highest priority: testing pediatric patients with hypotonia, developmental delay, movement disorders, and oculogyric crises; inclusion of 3-OMD dosing on DBS in neonatal screening programs; development of a clinical score to support patients' selection for 3-OMD screening; among atypical phenotypes based on clinical characteristics of Italian patients: testing patients with intellectual disability and parkinsonism-dystonia. *Discussion*. Clusters of symptoms and signs can be used to prioritize testing with 3-OMD DBS. A clinical score was rated as highly relevant for the patient's selection. The inclusion of 3-OMD dosing in newborn screening programs was advocated with high clinical priority.

## 1. Introduction

Aromatic l-amino acid decarboxylase (AADC) deficiency (AADCd) is a rare autosomal recessive neurometabolic disease caused by pathogenic variants in the dopa decarboxylase (*DDC*) gene, located at the short arm of chromosome 7, encoding the AADC enzyme [1]. Lack of the AADC enzyme leads to a severe combined deficiency of dopamine, serotonin, noradrenaline, and adrenaline [2].

Currently, there are 581 known variants of the *DDC* gene [3], of which the largest group is represented by missense variants [4]. Approximately 90% out of 151 genotypes had variants classified as pathogenic or likely pathogenic according to the ACMG/AMP/ACGS criteria, while 7% had one VUS allele and 3% had two VUS alleles [3].

Key clinical symptoms of AADCd include hypotonia, movement disorders, oculogyric crises, developmental delay, and autonomic symptoms. Patients typically present within the first few months of life [2, 5]. Alongside the “classic” severe phenotype, affecting 70% of patients, with profound motor impairment and minimal voluntary movement, a minority of patients have mild motor impairment and are able to walk independently [6]. Atypical presentations include early myasthenia-like manifestations and early-onset parkinsonism [7], but also syndromic intellectual disability with marfanoid habitus, craniofacial dysmorphisms, chronic diarrhea, and progressive kyphoscoliosis [8]. The majority of patients with AADC deficiency are at a high risk of premature death in early childhood due to complications from the disease, severe motor impairment, and oculogyric crises [9, 10].

Due to similarities in clinical presentation with other conditions (e.g., cerebral palsy or epilepsy), patients with AADCd are often undiagnosed or misdiagnosed [5, 6, 11, 12]. In fact, although most of the neurological and extraneurological symptoms and signs are frequently represented in each patient with AADCd [5], these are not specific, and correct identification of patients and timely diagnosis are challenging.

This is also negatively affected by the complexity of the diagnostic work-up. In order to correctly classify and thoroughly characterize affected individuals, the consensus recommendations published in 2017 established that two out of three of the following core diagnostic tests should be positive to confirm AADCd diagnosis [2, 11]:

- cerebrospinal fluid (CSF) neurotransmitter metabolites profile: low 5-hydroxyindoleacetic acid (5-HIAA), homovanillic acid (HVA) and 3-methoxy-4-hydroxyphenylglycol (MHPG) and high 3-O-methyldopa (3-OMD), L-dopa, and 5-hydroxytryptophan (5-HTTP) with normal pterins
- single *DDC* gene or genetic panel testing detecting pathogenic variants
- plasma enzyme assay (low levels of AADC enzyme activity in plasma)

Additional investigations can be used to screen for AADCd, including dried blood spot (DBS) [2, 11, 13], detecting high 3-OMD levels, also observed in pyridoxal 5′phosphate oxidase (PNPO) deficiency, which nevertheless has a different clinical presentation with severe epileptic encephalopathy and urinary testing detecting increased vanillactic acid levels, although its use is more limited because normal levels do not rule out AADCd [2].

In light of the availability of a screening test and based on the approval of gene therapy with eladocagene exuparvovec for patients with confirmed AADCd aged at least 18 months and a severe phenotype by the European Commission (EU/1/22/1653/001) and marketing authorisation by the Medicines and Healthcare Products Regulatory Agency (MHRA) in Great Britain, there is an urgent need to increase awareness on this condition among community-based healthcare professionals, with the objective not to miss diagnoses. This objective has already emerged as one of the top fields of intervention to improve the correct management of patients with AADC deficiency in a previous Delphi consensus among Italian experts on AADCd diagnosis and management [14].

To further pursue such an objective, this new project is aimed at focusing on the typical and atypical clinical symptoms and signs suggestive of AADCd in order to provide guidance on the correct selection of patients to be screened with 3-OMD on DBS and, if positive, to be sent for AADCd confirmatory tests.

## 2. Materials and Methods

As solid (levels 1-4) evidence on the clinical and laboratory criteria for 3-OMD DBS testing is currently lacking, the Delphi method was considered the best way to reach a consensus among Italian experts on AADCd (level of evidence: 5, expert opinion).

### 2.1. Delphi Method

The Delphi method is an investigation method based on the replies given by a panel of experts to a standardized questionnaire in order to reach the best consensus and provide recommendations or define standards in the absence of direct and sound evidence on a given topic [15–17].

It requires the iterative administration of a series of questions in order to elicit participants' opinions and also to promote a debate on a specific research topic.

Participants are experts in the field, and their numbers can range between a few and hundreds of persons.

The statements are the core elements of a Delphi survey, enabling the collection of the experts' opinions. Statements can either be single or divided into different items, to which the group of experts can anonymously and freely express their level of agreement or disagreement by using a scale.

In this paper, the Likert scale will be used, which is divided as follows: 1 = complete disagreement; 2 = disagreement; 3 = uncertain; 4 = agreement; 5 = complete agreement. If the combination of answers equalling 1 and 2 is higher than 80%, a negative consensus is reached. Conversely, if the combination of answers equalling 3, 4, and 5 is higher than 80%, a positive consensus is reached. If the total of 1-2 or 3-4-5 answers is lower than 80%, no consensus is reached. In the prioritization phase, 1 stands for “not relevant at all,” 2 for “little relevant,” 3 for “quite relevant,” 4 for “highly relevant,” and 5 for “extremely relevant.”

Results are collected and analyzed to establish how many statements have reached a positive or negative consensus and how many did not reach such a consensus. The process ends when an agreement has been reached on all of the discussed topics [15, 17].

### 2.2. Criteria for Expert Panel Profiling

Experts were selected based on their clinical/research/teaching experience, on membership of scientific societies, and position (i.e., being head of unit and head of department), balancing different specialties, academic and nonacademic positions, gender, and geographical distribution. The panel of experts was selected by asking each member of the steering committee to propose 2-3 experts, of whom no more than one worked in the same institution as the proposing member [16].

### 2.3. Prioritization Phase

After the validation of 13 statements, which was based on careful literature review and discussion within the steering committee, the experts selected by the steering committee, who did not take part in the first phase, voted online through a secure web platform. Participation was voluntary. To reduce the risk of bias or influence by other specialists' opinions, all answers were collected anonymously. Panelists were asked to prioritize statements, which were then listed according to their level of clinical priority.

### 2.4. Online Discussion of Results

In a second, online meeting, the steering committee analyzed the results of the Delphi survey. A series of consensus-based recommendations were finalized.

Responses from the experts were summarized descriptively (numbers, percentages) and graphically to identify outliers. In the prioritization phase, results for each statement were calculated as weighted average scores.

As the study does not involve patient data management, it did not require ethical approval. The involved experts were informed of the study's objectives and the possibility of publishing its results.

## 3. Results and Discussion

On 20^th^ July, the first meeting of the steering committee was held in Bologna (Italy), where the 14 experts met and discussed this project's aims, the main literature review findings, and the draft containing a proposal for statements and items and voted them as previously illustrated.

### 3.1. Steering Committee

The steering committee included 14 Italian medical doctors with special expertise in treating patients with AADCd.

### 3.2. Selection of Delphi Questionnaire Topics

Based on a careful review of the literature performed before the first meeting (July 2022), the steering committee selected the following topics:

1. Typical phenotype—clusters of symptoms and signs prompting consideration of 3-OMD dosing on DBS
2. Atypical phenotype—clusters of symptoms and signs possibly prompting consideration of 3-OMD dosing on DBS
3. Laboratory criteria for 3-OMD dosing on DBS
4. Usefulness of a clinical scoring system for patients' selection for 3-OMD dosing on DBS
5. Inclusion of 3-OMD dosing on DBS in neonatal screening

### 3.3. Delphi Participants

The voting panel was composed of 29 medical doctors (21 women), with clinical experience in managing patients with AADCd, divided as follows: 13 pediatricians, 12 child neuropsychiatrists, 3 neurologists, 2 biochemists, and one clinical geneticist. 16 (55%) came from northern Italy, 8 (28%) from southern Italy, and 5 (17%) from central Italy. Age groups and professional experience were homogeneously distributed among responders, as 14 out of 29 panel members were older than 50 years of age and 14 out of 29 had more than 20 years of clinical experience. Responders represented 13 Italian or European scientific societies, covering different medical disciplines (SIMMESN: 10; SINP: 9; SIP, LICE, and SSIEM: 6 each; SINPIA: 5; EPNS, Neumologia Pediatrica, and SIN: 3 each; SIMD, SIGU, International Parkinson and Movement Disorder Society, and LIMPE-DISMOV: 1 each).

### 3.4. Ranking of Statements Based on Weighted Average Scores

1. Statement 1. Regarding the identification of pediatric patients to be offered, 3-OMD screening for at-risk populations, hypotonia, developmental delay, movement disorders, and oculogyric crises should be considered as the most common symptoms and signs (weighted average score: 4.86)
2. Statement 14. Considering the easiness of AADCd screening with 3-OMD dosing, I would consider its inclusion in neonatal screening programs as useful (weighted average score: 4.59)
3. Statement 13. I would consider implementing a clinical score to support patients' selection for 3-OMD screening in at-risk populations as useful (weighted average score: 4.34)
4. Statement 7. Based on the clinical characteristics of patients so far identified in Italy, I would consider other clinical phenotypes (among those proposed) to be viewed as indicative in the choice to test a patient with 3-OMD screening for AADCd: intellectual disability and parkinsonism-dystonia (weighted average score: 3.9)
5. Statement 2. Regarding the identification of pediatric patients to undergo 3-OMD testing, I would rate it as important considering the following clusters of symptoms and signs as the most common: developmental delay and movement disorder with or without extraneurological symptoms (weighted average score: 3.76)
6. Statement 3. Regarding the identification of pediatric patients to undergo 3-OMD testing, I would rate it as important to consider the following clusters of symptoms and signs as the most common: developmental delay, neurovegetative symptoms, and pseudomyasthenic features (weighted average score: 3.62)
7. Statement 4. Regarding the identification of pediatric patients to undergo 3-OMD testing, I would rate it as important to consider the following clusters of symptoms and signs as the most common: intellectual disability, movement disorder, gastroenteric symptoms, and/or hypoglycemic episodes (weighted average score: 3.59)
8. Statement 8. Based on the clinical characteristics of patients so far identified in Italy, I would consider other clinical phenotypes (among those proposed) to be viewed as indicative in the choice to test an at-risk patient with 3-OMD screening for AADCd: neurovegetative symptoms, hypoglycemic episodes, psychiatric symptoms, and sleep disorder (weighted average score: 3.48)
9. Statement 12. In adolescent and adult patients with intellectual disability and movement disorder, among the following clinical or laboratory criteria I would consider as useful in order to identify patients to undergo 3-OMD testing: gastroenteric symptoms and neurovegetative symptoms (weighted average score: 3.31)
10. Statement 5. Based on the clinical characteristics of patients so far identified in Italy, I would consider other clinical phenotypes (among those proposed) to be viewed as indicative in the choice to test a patient with 3-OMD for AADCd: intellectual disability and pseudomyasthenic features (weighted average score: 3.28)
11. Statement 10. In adolescent and adult patients with intellectual disability and movement disorder, among the following clinical or laboratory criteria I would consider as useful in order to identify patients to undergo 3-OMD testing: fatigability and eyelid ptosis (weighted average score: 3.24)
12. Statements 9 and 11. In adolescent and adult patients with intellectual disability and movement disorder, among the following clinical or laboratory criteria I would consider as useful in order to identify patients to undergo 3-OMD testing: hyperprolactinemia and hypoglycemic episodes (weighted average score in both cases: 3.1)
13. Statement 6. Based on the clinical characteristics of patients so far identified in Italy, I would consider other clinical phenotypes (among those proposed) to be viewed as indicative in the choice to test a patient with 3-OMD testing for AADCd: syndromic intellectual disability (dysmorphisms, marfanoid habitus) and neurovegetative symptoms (weighted average score: 3.03)

The main clinical scenarios in which to consider 3-OMD testing, according to age and presence of typical versus atypical presentations, are summarized in Table 1.

AADCd is a rare neurotransmitter disorder, most often leading to a severe and complex neurological and extraneurological phenotype. Two key features of AADCd make its diagnosis challenging: first, its marked clinical heterogeneity, with typical and atypical symptoms; and second, its rarity. These issues determine a high risk for missed diagnoses, which, with the advent of gene therapy, we urgently need to abate.

A second issue is represented by a diagnostic delay. Among adult patients, this can reach 32 years [2, 6]. Such a delay, in light of outcome data in treated patients [18, 19], suggesting better results in those treated earlier [19, 20], makes early diagnosis imperative. Although confirmatory biochemical and molecular diagnosis can be considered as invasive and might not be available at every medical facility, 3-OMD dosing on DBS, an easy-to-use and reliable screening test, should facilitate the initiation of the diagnostic pathway if AADCd is suspected.

At this stage, high priority should be given to projects aiming to increase awareness of AADCd among practitioners, especially those working in community services. In order to do so, it is critical to first identify the most appropriate targets for these initiatives (such as local pediatricians or general practitioners) and subsequently select the most effective ways to deliver clinically meaningful, targeted information to promote 3-OMD patients' screening among the right at-risk populations. In other words, it is crucial to promote sensitive but also specific enough recommendations, providing a good balance between promoting patients' inclusion in at-risk populations' screening programs and the risks of an alarming communication resulting in unnecessary testing of patients with nonspecific extraneurological symptoms or signs, which can be part of the atypical AADCd spectrum but are also very common in the general pediatric population (i.e., diarrhea). In fact, in general, there is a high prevalence [5, 6] of neurological symptoms and signs in AADCd, which should first prompt clinical suspicion [2, 5].

Accordingly, the use of a clinical score to select suspected AADCd children for patients' screening by 3-OMD dosing was rated as highly to extremely relevant by our panel of experts. In fact, the selection of the correct clusters of symptoms and signs is obviously critical, but clinical heterogeneity and the association between neurological and extraneurological nonspecific symptoms and signs might make this diagnosis challenging. Based on previous preliminary experiences in Italy [21], it seems that the inclusion of broad, nonspecific neurodevelopmental symptoms and signs might not be a cost-effective strategy for implementing a diagnostic rate. Possibly, this might especially apply to countries with extremely low incidences of the disorder. We believe that these preliminary results further strengthen the potential usefulness of the approach to AADCd testing with 3-OMD reported in this paper.

In light of the lack of clear-cut evidence, we provide guidance based on the results of our Delphi survey among Italian experts in the care of patients with AADCd, prioritizing the clinical phenotypes to be screened within these clinical scenarios: typical phenotypes in pediatric patients, atypical phenotypes in teenagers and adult patients, and the presymptomatic phase (newborn screening).

Within typical presentations, in line with the abovementioned literature-based premises, the panel of experts gave the highest priority to testing pediatric patients presenting with hypotonia, developmental delay, movement disorders, and oculogyric crises, considered as the most common cluster of symptoms and signs in AADCd, followed by developmental delay and movement disorder, with or without extraneurological symptoms; developmental delay, neurovegetative symptoms, and pseudomyasthenic features; and, finally, intellectual disability, movement disorder, gastroenteric symptoms, and/or hypoglycemic episodes. All of these phenotypic clusters were rated as highly to extremely relevant, in line with previous clinical research data [1, 2, 5, 6, 22–25].

Within atypical phenotypes, based on data gathered on patients with AADCd in Italy, the experts' panel prioritized intellectual disability and parkinsonism-dystonia as the first phenotype to be considered for testing with 3-OMD, followed by neurovegetative symptoms, hypoglycemic episodes, psychiatric symptoms, and sleep disorder, and intellectual disability plus pseudomyasthenic features (all with average-to-high relevance). Syndromic intellectual disability (dysmorphisms, marfanoid habitus) with neurovegetative symptoms was the least prioritized atypical phenotypic cluster (average relevance). These results nicely reflect literature data reporting on the long-term follow-up of nine Italian patients showing intellectual disability, parkinsonism-dystonia, and psychiatric disorders in all cases [26]. The Consensus Guideline published in 2017 was also rated as “conditional,” considering AADCd in patients with autonomic features even without movement disorder [2]. Some of the described Italian patients exhibit pseudomyasthenic features (ptosis, fatigability, and diurnal variation of symptoms) [7, 8], which are reported in approximately one-quarter of described patients [5], while dysmorphic features have been rarely reported (4 Italian patients out of 9 in [8, 26]).

For the last clinical scenario regarding an adolescent or adult patient with intellectual disability and movement disorder, the additional clinical or laboratory criteria sustaining eligibility for 3-OMD testing include gastroenteric and neurovegetative symptoms as the most relevant cluster, followed by fatigability and eyelid ptosis, hyperprolactinemia, and hypoglycemic episodes. From our recent literature review, gastrointestinal symptoms have been reported in 19% of patients and hypoglycemic episodes in 10% [5]. Prolactin can be increased in AADCd, although normal levels do not exclude diagnosis [2]. Thus, the panelists' position regarding clinical symptoms and signs and additional laboratory findings gives operational guidance for patients' screening with 3-OMD of teenagers and adults in selected cases, although with lower priority compared to previous statements, possibly reflecting the ancillary role, lower specificity, and/or frequency of the proposed clinical and laboratory features in AADCd presentation. With this in mind, it is important to also highlight that further assessment of 3-OMD DBS concentrations in this age group has been recommended by some researchers in order to strengthen data on age-specific cut-off values because 3-OMD levels negatively correlate with age [13].

A further and critical result of this Delphi survey is that the panel of experts rated the inclusion of 3-OMD dosing on DBS in newborn screening programs as useful and gave high-to-extreme relevance to this issue. Our panel's opinion is in agreement with the literature data, highlighting that due to its easiness, reliability, and feasibility [1, 13, 27] and the potential benefits of promptly administering a disease-modifying therapy [19, 20], a diagnosis should be best reached in the presymptomatic phase [13]. A pilot study on the use of 3-OMD DBS as a screening tool in newborns was performed in Taiwan on 127,987 subjects, resulting in 4 diagnoses. The positive predictive value was 100%, and the false-positive rate was zero. No false-negative cases emerged during the study period [1]. A second pilot study using a different flow-injection analysis tandem mass spectrometry demonstrated the feasibility of introducing 3-OMD dosing in the expanded newborn screening in Italy [27]. A third paper established a new tandem mass spectrometry method to analyse 3-OMD in DBS with low intra- and interessay variability [13]. Scientific societies will have a crucial role in promoting the inclusion of 3-OMD in newborn screening programs.

## 4. Conclusions

By using the Delphi method, different clusters of clinical symptoms and signs and laboratory data were prioritized and selected in order to guide the correct identification of patients to be offered 3-OMD testing by community services-based pediatricians and general practitioners. Additional projects on which the panel of experts agreed include realizing a clinical score to help patients' selection and involving the pertinent medical scientific societies to work on the inclusion of 3-OMD DBS in newborn screening programs.

## Figures and Tables

**Table 1:** List of statements according to age and presence of atypical findings.

Clinical scenarios	Agreement on level of priority
Neonatal period and 3-OMD screening	
Statement 14. Considering the easiness of AADCd screening with 3-OMD dosing, I would consider its inclusion in neonatal screening programs as useful	Weighted average score: 4.59
Statement 13. I would consider implementing a clinical score to support patients' selection for 3-OMD screening in at-risk populations as useful	Weighted average score: 4.34
Pediatric age	
Statement 1. Regarding the identification of pediatric patients to be offered 3-OMD screening for at-risk populations, hypotonia, developmental delay, movement disorders, and oculogyric crises should be considered as the most common symptoms and signs	Weighted average score: 4.86
Statement 2. Regarding the identification of pediatric patients to undergo 3-OMD testing, I would rate it as important considering the following clusters of symptoms and signs as the most common: developmental delay and movement disorder with or without extraneurological symptoms	Weighted average score: 3.76
Statement 3. Regarding the identification of pediatric patients to undergo 3-OMD testing, I would rate it as important to consider the following clusters of symptoms and signs as the most common: developmental delay, neurovegetative symptoms, and pseudomyasthenic features	Weighted average score: 3.62
Statement 4. Regarding the identification of pediatric patients to undergo 3-OMD testing, I would rate it as important to consider the following clusters of symptoms and signs as the most common: intellectual disability, movement disorder, gastroenteric symptoms, and/or hypoglycemic episodes	Weighted average score: 3.59
Teenage years—adulthood	
Statement 12. In adolescent and adult patients with intellectual disability and movement disorder, among the following clinical or laboratory criteria I would consider as useful in order to identify patients to undergo 3-OMD testing: gastroenteric symptoms and neurovegetative symptoms	Weighted average score: 3.31
Statement 10. In adolescent and adult patients with intellectual disability and movement disorder, among the following clinical or laboratory criteria I would consider as useful in order to identify patients to undergo 3-OMD testing: fatigability and eyelid ptosis	Weighted average score: 3.24
Statements 9 and 11. In adolescent and adult patients with intellectual disability and movement disorder, among the following clinical or laboratory criteria I would consider as useful in order to identify patients to undergo 3-OMD testing: hyperprolactinemia and hypoglycemic episodes	Weighted average score in both cases: 3.1
Atypical phenotypes	
Statement 7. Based on the clinical characteristics of patients so far identified in Italy, I would consider other clinical phenotypes (among those proposed) to be viewed as indicative in the choice to test a patient with 3-OMD screening for AADCd: intellectual disability and parkinsonism-dystonia	Weighted average score: 3.9
Statement 8. Based on the clinical characteristics of patients so far identified in Italy, I would consider other clinical phenotypes (among those proposed) to be viewed as indicative in the choice to test an at-risk patient with 3-OMD screening for AADCd: neurovegetative symptoms, hypoglycemic episodes, psychiatric symptoms, and sleep disorder	Weighted average score: 3.48
Statement 5. Based on the clinical characteristics of patients so far identified in Italy, I would consider other clinical phenotypes (among those proposed) to be viewed as indicative in the choice to test a patient with 3-OMD for AADCd: intellectual disability and pseudomyasthenic features	Weighted average score: 3.28
Statement 6. Based on the clinical characteristics of patients so far identified in Italy, I would consider other clinical phenotypes (among those proposed) to be viewed as indicative in the choice to test a patient with 3-OMD testing for AADCd: syndromic intellectual disability (dysmorphisms, marfanoid habitus) and neurovegetative symptoms	Weighted average score: 3.03

## Data Availability

The data that support the findings of this study can be made available from the corresponding author by reasonable request.
